# Current and future opportunities for the management of primary biliary cholangitis

**DOI:** 10.3389/fgstr.2023.1241901

**Published:** 2023-12-04

**Authors:** Sandra Naffouj, Jennifer Wang

**Affiliations:** Division of Gastroenterology and Hepatology, Department of Medicine, University of Illinois, Chicago, IL, United States

**Keywords:** primary biliary cholangitis, treatment, ursodeoxycholic acid, obeticholic acid, fibrates, peroxisome proliferator-activated receptors

## Abstract

Primary biliary cholangitis (PBC) is a rare immune-mediated chronic cholestatic liver disease that can progress to liver fibrosis and, ultimately, cirrhosis if left untreated. Since the pathogenesis of PBC is not well understood, curative therapies have yet to be established. Ursodeoxycholic acid (UDCA), the standard of care treatment for PBC, has been proven to reduce disease progression and improve transplant-free survival. However, one third of patients have no response or partial biochemical response to UDCA and are at increased risk for disease progression. In such cases, second-line therapy with obeticholic acid (OCA) or peroxisomes proliferator-activated receptors (PPARs) should be considered in conjunction with UDCA. In this review article, we aim to provide an overview of the most recent data on PBC treatment in patients with inadequate response to UDCA, as well as novel therapies in the early stages of development.

## Introduction

Primary biliary cholangitis (PBC) is an uncommon chronic cholestatic liver disease, characterized by immune-mediated inflammatory destruction of the small and medium intra-hepatic bile ducts (1). It predominantly affects women, and its peak incidence occurs in the fourth and fifth decades of life. If left untreated, it can progress to liver fibrosis, and ultimately, cirrhosis with its related complications (2). Since the pathogenesis of PBC is not very well understood, curative therapies have yet to be established, and the focus has been on preventing disease progression and ameliorating symptoms. Ursodeoxycholic acid (UDCA) is the standard of care and recommended for all patients with PBC by the American Association for the Study of Liver Disease (AASLD) and the European Association for the Study of the Liver (EASL) (3, 4). It has been shown to remarkably improve the progression of PBC as well as clinical outcomes (5, 6). The recommended dose is 13-15 mg/kg of body weight per day. It is a hydrophilic bile acid with an excellent safety profile, even in pregnancy (7). Additionally, a recent retrospective study showed that UDCA use as a preventative therapy post-liver transplant in PBC patients was associated with decreased risk of PBC recurrence, graft loss, and all-cause mortality (8). However, it has been estimated that 30-40% of patients with PBC have no response or partial biochemical response to UDCA and are at high risk for disease progression, highlighting the necessity for novel therapies in the management of PBC (9). In such cases, a second-line treatment is recommended in conjunction with UDCA (10). In this review, we aim to summarize the most recent evidence on PBC therapies in patients with inadequate response to UDCA as well as developing therapies on the horizon.

## Prognostic tools for PBC

It is imperative to risk stratify PBC patients and identify high-risk groups who may progress to cirrhosis with its associated complications. Poor prognostic factors prior to starting treatment are young age at disease onset (<45 years), male gender, advanced fibrosis, and abnormal total bilirubin (T. bili) and albumin (4). Additionally, stratifying patients based on their biochemical response to UDCA after one year of therapy offers a readily available measure to identify high-risk groups that may benefit from additional treatment. The two most used prognostic models are GLOBE and UK-PBC scores (11, 12). Biochemical markers including alkaline phosphatase (ALP) and T. bili have been shown to independently predict outcomes in PBC patients, including need for liver transplantation (LT) and death (13). Therefore, they have been widely used as surrogate markers for disease outcomes in clinical trials given rarity of the disease and its slow progressive nature (14). Fibrosis assessment in PBC patients also provides an important prognostic factor irrespective of the biochemical response, suggesting that fibrosis staging should be incorporated into risk stratification models (15). Considering all these findings, patients who do not achieve targeted biochemical response after one year of UDCA and those with advanced fibrosis are at high risk for disease progression and should be evaluated for second-line treatments. A summary of the most recent clinical trials on novel therapies in PBC is demonstrated in **Table 1**.

**Table 1:** Summary of the most recent clinical trials on novel therapies in PBC.

Medication	Trial/duration	Subjects’ Groups	Outcomes/primary end point	Considerations
**Obeticholic acid (OCA)** ** **	POISE RCT^(16)^ 12 months	. OCA 10 mg group + UDCA . OCA 5-10 mg group + UDCA . Placebo + UDCA	. ALP <1.67 ULN with 15% reduction from baseline and normal T.bili	. Pruritis is the most common side effect . No change in fibrosis . OCA is contraindicated in advanced or decompensated cirrhosis and biliary obstruction
**Bezafibrate (pan-PPAR agonist)**	BEZURSO RCT^(26)^ 24 months	. Bezafibrate 400 mg daily + UDCA . Placebo + UDCA	Biochemical response (normal Tbili, ALP, aminotransferase, albumin, prothrombin index)	. Improved pruritis, fatigue, and liver fibrosis . Limited use in advanced cirrhosis . Concerns regarding kidney function, liver enzyme elevation, and myalgias
**Bezafibrate (pan-PPAR agonist)**	FITCH RCT^(27)^ 21 days	. Bezafibrate 400 mg daily . Placebo Patients with moderate-severe pruritis from cholestatic disease (VAS>5 of 10)	. >50% reduction of pruritis	. Short term study with unclear long-term safety . Increase in serum creatinine in 3% of treated patients
**Elafibranor (dual-PPAR agonist)** ** **	Phase II RCT^(29)^ 12 weeks	. Elafibranor 80 mg . Elafibranor 120 mg . Placebo	. Primary: % change in ALP . Composite end point: ALP <1.67 ULN, ALP reduction >15%, Tbili < 1 ULN	. Improved pruritis . Improved gamma-glutamyl transferase (GGT), high-sensitivity C-reactive protein . Side effects: nausea, diarrhea, fatigue, headache . Phase III RCT ELATIVE is underway; it will include markers of fibrosis
**Saroglitazar(dual-PPAR agonist)** ** **	Phase II RCT^(31)^ 16 weeks	. Saroglitazar 4 mg . Saroglitazar 2 mg . Placebo	. Endpoint: ALP reduction	. 4 patients in the 4 mg group developed elevated liver enzymes (DILI), withdrew from study . Further studies at 1 mg and 2 mg are underway
**Seladelpar (PPAR-delta agonist)** ** **	Phase II open label study^(32)^ 52 weeks	.Seladelpar 5 mg (n=53) .Seladelpar 10 mg (n=55) .Seladelpar 2 mg (N=11) 12-week dose-ranging period 40-week extension period	. Primary endpoint: ALP change at week 8 . Secondary endpoints: biochemical response (ALT, AST, GGT, LDL, TG, C4, FGF-19) . Pruritis (VAS score)	. The criteria for dose increase were not standardized . Improved pruritis . Improved lipid profile . Phase III trial (RESPONSE) seladelpar 10 mg vs. 5 mg vs. placebo 12 months
**Budesonide**	Phase III RCT^(36)^ 36 months	. Budesonide 9 mg daily + UDCA (12-16 mg/kg/day) . Placebo + UDCA (12-16 mg/kg/day)	. Histological improvement and preventing fibrosis progression (not achieved) . POISE end point of biochemical response were reached in treatment group	. Budesonide may improve biochemical markers in non-cirrhotic PBC patients . Consider steroid related side effects
**NOX Inhibitor**	Phase II RCT^(48)^ 24 weeks	. Setanaxib 400 mg daily + UDCA . Setanaxib 400 mg BID + UDCA . Placebo	. Primary endpoint: change from baseline in GGT . Secondary endpoint: change from baseline in ALP, liver stiffness via elastography, and fatigue.	. Preliminary results suggest possible anti-cholestatic and anti-fibrotic effects

## Obeticholic acid

Obeticholic acid (OCA) is a synthetic bile acid that acts as a potent farnesoid X receptor (FXR) agonist, impairing bile acid synthesis and promoting bile acid secretion. It is the only approved second-line therapy in adult PBC patients in the US, recommended to be used in conjunction with UDCA in partial responders or as monotherapy in those who are intolerant to UDCA (3, 4). The medication was approved by the Food and Drug Administration (FDA) and European Medicines Agency (EMA) based on results of the POISE trial in 2016, showing its efficacy in improving biochemical markers in high-risk subgroups (16). The trial was a 12-month, double-blinded, placebo-controlled phase III trial that included 217 patients who were partial responders or intolerant to UDCA. Subjects were randomized to receive 10 mg of OCA, 5 mg of OCA with up-titration to 10 mg, or placebo in addition to UDCA. A small group of patients received OCA as monotherapy due to intolerance to UDCA. The primary endpoint of achieving ALP reduction and normal T. bili occurred more frequently in both OCA groups (46 – 47%) compared to the placebo group (10%). There was no difference in fibrosis staging between the groups at the end of the study, and dose-dependent pruritis was the most common side effect related to OCA. The recommended starting dose is 5 mg, which can be increased to 10 mg if reduction in ALP and/or bilirubin does not occur within 3 months (17). OCA can also be started earlier in the course for patients who are intolerant to UDCA, more commonly due to GI symptoms such as unexplained diarrhea (18). In 2021, the FDA updated the label on OCA, restricting its use in patients with advanced cirrhosis defined as current or prior episodes of decompensation events or with compensated cirrhosis who have evidence of portal hypertension. The AASLD then published an updated practical guidance statement reporting the contraindicated use of OCA in advanced cirrhosis and recommending cautious use in select patients with compensated cirrhosis and without features of portal hypertension with close monitoring (19). Similarly, the EMA updated the OCA label in 2022 prohibiting its use in decompensated cirrhosis including Child-Pugh Class B and C (20).

Real-world data continue to emerge, demonstrating the safety and efficacy of OCA on clinical outcomes such as persistent improvement in ALP, stabilization of bilirubin, fibrosis, and transplant-free survival (21–23). For example, the 5-year open-label extension study of the POISE trial showed that long-term OCA treatment was associated with improved histological features, including fibrosis and ductular injury (22). Interim analyses from the same study confirmed the efficacy and safety of OCA at three years with continued improvement in ALP levels and stabilization of T. bili with no serious adverse events reported. Pruritis and fatigue remained the two most common adverse events (23). Additionally, patients in the POISE group had significantly better transplant-free survival when compared to the Global PBC control group at the 6-year mark with hazard ratios (HRs) of 0.29 (95% CI, 0.10-0.83) (24).

A phase 4 clinical trial is ongoing to evaluate the effect of OCA on long-term clinical outcomes (COBALT NCT02308111). In addition to OCA, newer FXR agonists are under investigation in PBC treatment, such as cilofexor (NCT02943447) and ASC 42 (NCT05190523).

## Peroxisomes proliferator-activated receptors

Fibrates are a class of hypolipidemic agents that act on the peroxisomes-proliferator-activated receptors (PPARs), a transcription factor involved in fat catabolism and inflammatory response, resulting in decreased bile acid synthesis in the liver, detoxification of bile acids, and increased bile acid output as well as suppressing inflammation (25). Fenofibrate is the only fibrate that is FDA-approved in the United States (US) for dyslipidemia and primary hypercholesterolemia, with ongoing phase II clinical trials to assess its efficacy in the management of PBC with and without UDCA (NCT01141296 and NCT00575042).

### Bezafibrate

Bezafibrate, a pan-PPAR agonist, is the third approved drug outside the US after UDCA and OCA based on the result of BEZURSO study. This double-blind, placebo-controlled phase III trial enrolled 100 PBC patients with inadequate response to UDCA defined by the Paris-2 criteria (i.e., a serum level of ALP or AST >1.5 times the ULN or an abnormal T.bili after 6 months or more of treatment, excluding patient with T. bili level >3 mg/dl) (26). Patients were randomized to receive 400 mg of bezafibrate or placebo in addition to UDCA for 24 months. The primary endpoint defined as a complete biochemical response (normal levels of T.bili, ALP, aminotransferase, albumin, and prothrombin index) was met in 31% of patients in the treatment group compared to none in the placebo group. Normal ALP level was achieved in 67% of the patients in the bezafibrate group compared to 2% in the placebo group. Additionally, parallel improvement in pruritis, fatigue, and liver stiffness was observed in the bezafibrate group. Features of portal hypertension and ALP level >2.53 the upper limit of normal (ULN), were independent predictors of treatment failure; thus, advanced cirrhosis and cholestasis are limiting factors to the use of bezafibrate. Although the rate of adverse events was not significant between the two groups, myalgia happened more frequently in the bezafibrate group, serum creatinine increased in 5% of patients in the treatment group compared to 3% in the placebo group, and finally ALT increased >5 times the ULN within the first 6 months of therapy initiation leading to treatment discontinuation, suggesting the importance of monitoring liver enzymes and kidney function after starting fibrates.

Bezafibrate also has beneficial effect on pruritis as demonstrated in the FITCH trial which included 79 patients with moderate to severe pruritis (visual analog scale >5) due to cholestatic liver disease including PBC, primary and secondary sclerosing cholangitis who were randomized to receive bezafibrate vs. placebo (27). 55% of patients in the PBC group achieved >50% reduction in pruritis compared to 13% in the placebo group (p=0.04) after 21 days. Furthermore, there was a significant reduction in ALP level associated with improved pruritis in the treatment group. No significant difference in creatinine change was noted between the two groups, concluding that bezafibrate is an effective short-term treatment of moderate to severe pruritis due to cholestatic diseases.

Although the data are limited on the long-term effect of fibrates on transplant-free survival in PBC patients with inadequate response to UDCA, Tanaka et al. showed in a large retrospective Japanese nationwide cohort study that bezafibrate-UDCA combination treatment was associated with reduction in all-cause mortality and need for LT compared to UDCA alone (HR 0.33, 95% CI 0.19-0.54, P<0.001) (28).

### Elafibranor

Elafibranor is another promising fibrate that is a dual-PPAR (alpha-delta) agonist. In a recent phase II double-blind RCT, 45 non-cirrhotic PBC patients with inadequate biochemical response to UDCA were randomized to elafibranor 80 mg, elafibranor 120 mg, or placebo (29). Patients who received either dose of elafibranor had a significant reduction in ALP level compared to the placebo group, achieving the composite endpoint of ALP <1.67-fold the ULN, a decrease of ALP >15%, and normalization of T.bili at 12 weeks. A favorable trend of improved pruritis was noted in patients who received elafibranor. Patients who received the 120 mg dose had a significant increase in sCr attributed to increased Cr release from muscle rather than a decrease in GFR since cystatin C level, a more accurate marker for GFR, remained unchanged. This study prepared the foundation for a larger prospective phase III trial, ELATIVE, that will determine the efficacy and safety of elafibranor as a second-line therapy for PBC (NCT04526665).

### Saroglitazar

Saroglitazar is a novel dual-PPAR (alpha-gamma) agonist. It is currently approved outside the US for treatment of diabetic dyslipidemia and hypertriglyceridemia with type 2 diabetes mellitus that is not controlled with statin therapy as well as non-alcoholic steatohepatitis (NASH) (30). Due to similarities in the mechanistic pathogenesis, the efficacy of saroglitazar is being evaluated in the management of PBC as a second-line therapy. Saroglitazar was effective in reducing ALP levels at doses of 2 mg and 4 mg compared to placebo in a recent phase II double bind RCT that included 37 PBC patients who are UDCA resistant or intolerant after 16 weeks of treatment. Of note, the study was terminated in 4 patients in the 4 mg group due to suspected drug-induced liver injury (DILI). Currently, investigations on doses of 1 mg and 2 mg are underway (31).

### Seladelpar

Seladelpar, a PPAR-delta agonist, was assessed in PBC patients who were non-responders or intolerant to UDCA, in a 52-week phase II, dose-ranging, open-label study randomizing patients in 1:1 ratio to seladelpar 5 mg/day (n=53) or 10 mg/day (n=55) or 2 mg/day (n=11) for 12 weeks (32). Doses could be titrated to 10 mg/day at or after 12 weeks of initiation. 21% of patients had compensated cirrhosis, and 71% had pruritis. The mean ALP levels were significantly reduced from baseline, and the reduction was dose-dependent; 23% in the 2 mg cohort, 35% in the 5 mg cohort, and 43% in the 10 mg cohort (p<0.005). Biochemical responses and further improvements were maintained until week 52. ALP normalization occurred in 31% of patients in the 10 mg cohort at week 12 and was durable at 33% through week 52. On the other hand, ALP normalization occurred in 0% and 11% of patients in the 2 mg and 5 mg groups at week 12, respectively, and in 9% and 13% at week 52, respectively. Furthermore, mean pruritis VAS scores improved from baseline in all three cohorts at week 12. Symptoms remained stable or continued to improve until the end of the study in a dose-dependent fashion. A pivotal phase III, randomized, placebo-controlled trial is ongoing to further assess the treatment effect and safety of Seladelpar (NCT04620733).

Despite convincing data on the efficacy of fibrates in the treatment of PBC, more data are needed on their safety in patients with advanced liver disease.

## Budesonide

Biliary cirrhosis in PBC occurs due to progressive cholestatic hepatitis caused by destructive cholangitis and interface hepatitis (33). The presence of autoimmune hepatitis features is often associated with increased serum aminotransferases and positivity of serum auto-antibodies, such as antinuclear antibodies (ANA) and anti-smooth muscle antibodies (ASMA). These autoimmune features are usually corticosteroid-responsive, similar to classic autoimmune hepatitis (9). Additionally, *in-vitro* data showed that budesonide and UDCA have a synergistic effect on increasing biliary secretion of bicarbonate with a protective effect on the biliary epithelium from toxic bile acids (34, 35). Based on these facts, Hirschfield et al. conducted a placebo-controlled randomized phase III trial on non-cirrhotic PBC patients who had histologically confirmed inflammatory activity based on the Ishak score and inadequate response to UDCA to evaluate the role of budesonide as an additional therapy to improve liver histology including inflammation and fibrosis (36, 37). Budesonide is not recommended in patients with cirrhosis due to the risk of portal vein thrombosis and uncontrolled systemic shunting of the medication (38). Patients were randomized to receive budesonide 9 mg daily in addition to UDCA 12-16 mg/kg/day vs. placebo and the same dose of UDCA. The study was underpowered due to recruitment challenges for the primary efficacy analysis. In the intention-to-treat analysis, there was no difference in the primary endpoint between the treatment and placebo groups at 36 months. However, the proportion of patients in the budesonide group who met the POISE endpoint of biochemical response (ALP <1.67 the ULN with a 15% reduction from baseline and normal T.bili) was higher compared to the placebo group. The two groups did not differ in the frequency of side effects or serious adverse events. Side effects such as hypertension, osteopenia, cataract, and weight gain were more common in the budesonide group and likely contributed to a higher number of discontinuations. Although this study did not show that budesonide improved histological outcomes in the UDCA-treated PBC patients, it showed that budesonide was associated with improved biochemical surrogate markers for disease progression, suggesting that it may be effective in PBC patients with marked histologic inflammatory activity. In the real-world setting, budesonide is rarely used as a second-line therapy for PBC as fewer biopsies are being performed to make the diagnosis, its contraindication in cirrhotic patients, and the high likelihood of achieving adequate response to the standard PBC therapies without immunosuppression even if overlap inflammatory features are present.

## The pipeline for future PBC therapies

There is a strong interest in developing newer therapies for PBC, and early initiation of combination therapy in high-risk patients is also under investigation. Most recently, a fixed dose of OCA and bezafibrate has received orphan drug designation by the FDA; this novel combination therapy may provide greater efficacy in bile acid synthesis and metabolism as it targets two different pathways in the pathogenesis of PBC. Two ongoing phase II trials (NCT04594694 and NCT05239468) are investigating a range of therapeutic doses of this combination treatment. An additional promising combination therapy is bezafibrate and UDCA in UDCA non-responders which showed significant improvement in biochemical markers, fibrosis, and inflammatory histological score at 5 years in 49 patients (39). However, larger randomized controlled trials are needed to confirm the efficacy of this combination. There are reports recommending the use of triple therapy (UDCA, OCA, and fibrates) in patients who fail to achieve biochemical response with UDCA and OCA or fibrates (40). Furthermore, CNP-104 is a novel proprietary system that combines disease-specific pathogenic antigens with pharmaceutical nanoparticles that mimics the routine removal of dying cells from the body. It is being studied in phase II double-blind placebo-controlled trial (NCT05104853). Several other promising therapies are in the early stages of development with a focus on both immune and non-immune pathways.

## Nor-urodeoxycholic acid

Nor-ursodeoxycholic acid (nor-UDCA) is a side-chain-shortened derivative of UDCA with special pharmacological features, making it a promising therapy for cholestatic liver disease (41). It has shown potent anti-cholestatic, anti-inflammatory and anti-fibrotic properties (42, 43). It is also distinguished by its intrahepatic enrichment, leading to a possible role in non-cholestatic metabolic and inflammatory liver disease (41). A recent phase II clinical trial showed that nor-UDCA was effective in reducing ALP levels within 12 weeks in a dose-dependent manner in primary sclerosing cholangitis (PSC) patients with an excellent safety profile (44). Phase III clinical trial is underway (NCT03872921). NorUDCA is currently undergoing phase II trial for PBC in Europe (EudraCT number: 2021-001431-56).

## NOX inhibitors

The nicotinamide adenine dinucleotide phosphate oxidases (NADPH oxidases, NOX) is the primary enzymatic source that regulates the production of reactive oxygen species (ROS), which have been implicated in a variety of disease processes including PBC (45). NOX1, NOX2, and NOX4 are expressed by the hepatocytes and play a significant role in mediating liver fibrosis and hepatocyte apoptosis (46). NOX4 additionally promotes the signaling of transforming growth factor beta (TGFβ-1), which contributes to fibrinogenesis in PBC (47). This suggests that selective NDAPH oxidase inhibitors, such as NOX4 inhibitors, might be a promising therapeutic agent. A recent phase II randomized trial evaluating the efficacy and safety of setanaxib, a NOX1/NOX4 inhibitor, in UDCA non-responsive patients suggested promising anti-cholestatic and anti-fibrotic effects for this agent as well as a role in improving fatigue warranting further evaluation in PBC patients with advanced fibrosis (48). An ongoing placebo-controlled double-blind clinical trial assessing biochemical response at 52 weeks in patients who are intolerant or have an inadequate response to UDCA with elevated liver stiffness (≥8.8 kPa) started in 2021 with an estimated completion date in 2024 (NCT05014672).

## Nudt1

T-cell dysfunction is another contributing factor to the pathogenesis of PBC (49). Cholangiocytes sustain cell-mediated autoimmune injury via clusters of autoreactive CD4+ CD8+ pyruvate dehydrogenase complex (PDC-E2) specific T cells in the liver (50). A recent study found that Nudt1 mediates the inappropriate expansion, hyperactivation, and long-term survival of CD 103+ cells, the primary autoreactive T cells demonstrating cytotoxicity against cholangiocytes in patients with PBC. Additionally, blocking Nudt1 resulted in ameliorating cholangitis in mice models (51). These investigations propose a new paradigm of developing highly selective immunomodulating therapies for treating PBC.

## Fibroblast growth factor agonists

Fibroblast Growth Factor 19 (FGF)-19 is an ileum-derived post-prandial enterokine that reduces bile acid synthesis in the hepatocytes via direct feedback, hence its potential role in PBC treatment (52). FGF-19 agonists have been shown to have anti-steatotic, anti-inflammatory, and anti-fibrotic properties (53). Aldafermin (NGM 282), an engineered FGF-19 analog, has achieved significant ALP reduction at 28 days in UDCA non-responders compared to placebo with an acceptable safety profile in a randomized, double-blind placebo-controlled phase II clinical trial (54). Further studies are needed to prove the durability of biochemical response and its effect on preventing fibrosis and liver decompensation and improving transplant-free survival.

## Janus kinase inhibitors

Barcitinib, a JAK 1 and JAK 2 inhibitor, is hypothesized to downregulate multiple cytokines involved in PBC pathogenesis. It was investigated in a clinical trial (NCT03742973) and showed efficacy in reducing ALP at 12 weeks in UDCA non-responders (55). However, the study was terminated due to a lack of enrollment. This mechanistic pathway remains a potential target for novel therapies in the future and further studies are warranted.

## Ustekinumab

The interleukin (IL)-12 pathway has been implicated in PBC pathogenesis. An open-label clinical trial evaluating the efficacy of ustekinumab did not result in a significant reduction in ALP and failed to establish the proof-of-concept for this therapy (56).

## Microbiome

Many studies have reported the link between gut dysbiosis and the pathophysiology of PBC, suggesting that gut microbiota may be a promising therapeutic target for PBC (57–59). Some of the proposed mechanisms through which the gut-liver axis contributes to PBC pathogenesis include the effect of dysbiosis on the intestinal mucosal immune system balance increasing gut permeability leading to increased bacterial translocation and abnormal immune system activation, which can cause tissue damage through molecular mimicry mechanisms and the lipopolysaccharide (LPS)-Toll-like receptor 4 (TLR 4) signaling pathway (60, 61). Another mechanism is the impaired bile-microbiota interactions, leading to poor bile acid metabolism and cholestasis (60). Based on these findings, probiotics and fecal microbiota transplantation (FMT) may provide new avenues for future PBC therapies by restoring healthy microbiota and repairing intestinal barrier function (62, 63). Current evidence is limited, and further studies are warranted to better understand and harness their therapeutic potential (64).

## Future directions

Despite the evidence of PBC being an autoimmune disease, the exact etiology remains unclear, making the establishment of surrogate endpoints difficult. Using histological response as a primary endpoint, like in the budesonide trial, remains challenging due to difficulties in recruitment. Gamma glutamyl transferase (GGT) is rising as an additional biochemical marker being assessed as a primary endpoint in clinical trials to predict clinical outcomes of liver transplantation and liver related death even in patients with low serum level of ALP (65). Furthermore, transient elastography-derived liver stiffness measurement has emerged as a surrogate marker for liver fibrosis in PBC, providing a non-invasive modality in monitoring disease progression and response to therapy over time and is being incorporated in recent trials. A large international retrospective multicenter study has shown that liver stiffness measurement by vibration-controlled transient elastography is an independent predictor for PBC clinical outcomes including liver transplantation and death with a measurement of 15 kilopascal (kPa) as a cut off for high-risk group (66).

Other issues that contribute to the challenges in the execution of clinical trials in PBC and setting hard clinical outcomes include the rare nature and slow progression of the disorder, hesitation of patients to stay on placebo as part of long-term outcome trials, which limit the achievement of adequate sample sizes and the ability to determine the efficacy of medications on transplant-free survival and mortality. Additionally, the significant heterogeneity in clinical trials limits the generalizability and interpretation of metanalysis results. Therefore, using a standard design in larger clinical trials and relying on proper endpoints is crucial in advancing clinical research in PBC.

## Conclusion

Therapies for PBC continue to progress with possible targets of normalizing liver enzymes, minimizing symptoms, and avoiding liver transplantation. A new paradigm is creating a personalized treatment approach depending on the individual risk factors, symptom burden, liver biochemistries, degree of fibrosis and potentially starting combination therapy with a step-down approach to prevent disease progression. To date, OCA is the only approved second-line therapy for PBC patients in the US. Fibrates have been shown to be effective in improving the biochemical surrogates for disease progression, pruritis, and potentially clinical outcomes compared to UDCA alone in high-risk patients, and its approval for PBC treatment is eagerly awaited. Multiple other promising fibrates are under investigation in phase II and III clinical trials as demonstrated in **Table 1**. The pipeline for additional second-line therapies is rapidly growing, with potential treatments targeting underlying mechanistic pathways in PBC pathogenesis, such as NOX inhibitors. This review demonstrates that recent advances have provided a better understanding of PBC pathogenesis, inspiring new treatment approaches beyond UDCA. However, unmet needs remain, especially for patients with high-risk features and symptomatic disease, emphasizing the importance of ongoing clinical trials to find more effective therapies.

## Author contributions

Both authors contributed to the study conception and design. SN drafted the initial portions of the manuscript. JW provided a critical revision of the manuscript. All authors contributed to the article and approved the submitted version.
